# Optimization of Medium Composition for High Cell Density Culture of *Bifidobacterium longum* HSBL001 Using Response Surface Methodology

**DOI:** 10.1002/mbo3.70027

**Published:** 2025-07-02

**Authors:** Hao Cheng, Jiangbin Liu, Liya Mei, Wei Liu, Fengxi Yang, Xiaojuan Ma, Yan Zhang, Youfa Xie, Yang Zhang, Yanxia Xiong

**Affiliations:** ^1^ Jiang Zhong Pharmaceutical Co. Ltd. Nanchang China; ^2^ State Key Laboratory of Food Science and Resources Nanchang University Nanchang Jiangxi China; ^3^ Key Laboratory of Taste Correction (Taste Masking) and Sensory Evaluation of Traditional Chinese Medicine Nanchang China

**Keywords:** anaerobic fermentation, *Bifidobacterium longum*, high cell density culture, medium optimization, response surface methodology

## Abstract

*Bifidobacterium longum* plays a critical role in the human gut and exhibits diverse probiotic functions. Achieving high‐density fermentation of *B. longum* largely depends on the composition of the culture medium and fermentation conditions. This study aimed to optimize the medium composition and fermentation parameters for *B. longum* HSBL001, using viable cell counts and optical density at 600 nm (OD600) as indicators. The goal was to improve biomass yield and support the development and industrial application of highly active probiotic preparations. The optimal medium composition and culture conditions were established using a combination of single‐factor experiments, the Plackett–Burman design, the steepest ascent method, and the central composite design. The optimized culture medium consisted of yeast extract (19.524 g/L), yeast peptone (25.85 g/L), arginine (0.599 g/L), glucose (27.36 g/L), MnSO_4_ (0.09 g/L), MgSO_4_ (0.8 g/L), Tween‐80 (1 g/L), l‐cysteine hydrochloride (0.24 g/L), and methionine (0.15 g/L). The optimal culture conditions included an initial pH of 7.0, 5% inoculum size, and incubation at 37°C, yielding a final viable cell count of 4.20 × 10^9^ colony‐forming units (CFU/mL). In a 3 L bioreactor, the viable cell count reached 1.17 × 10^10^ CFU/mL, which was 1.786 times higher than that achieved with the modified MRS medium. These findings demonstrate that the optimized medium and fermentation conditions are well‐suited for high‐density cultivation of *B. longum* HSBL001 and provide a basis for its industrial application.

## Graphical abstract

The optimal culture medium formula and fermentation process of *Bifidobacterium longum* HSBL001 were determined, and the fermentation level reached 1.17 × 10^10^ colony‐forming units/mL, which lays a foundation for the industrial application of *B. longum*.

## 1 Introduction

*Bifidobacterium* is among the earliest microbial genera to colonize the human intestinal tract, and its abundance and diversity are closely associated with overall health (He et al. 2022). Within this genus, *Bifidobacterium longum* is considered a key member of the human intestinal microbiota and is the most abundant species in the intestinal tract of infants (Mills et al. 2023). Currently, it is widely applied in the food, pharmaceutical, animal feed, and healthcare industries and is recognized both domestically and internationally as a safe microbial strain for food processing (Takeda et al. 2023; Xiao et al. 2022; McCarville et al. 2017). Several studies have demonstrated the health benefits of *B. longum*, including the alleviation of irritable bowel syndrome (Lewis et al. 2020), improvement of inflammatory bowel disease (Yao et al. 2021), relief of constipation (Russo et al. 2017), improvement of immune function (Finamore et al. 2019), support of cognitive development (Inoue et al. 2018), mitigation of atopic dermatitis (Navarro‐López et al. 2018), management of type 2 diabetes mellitus (K. Gao et al. 2023), promotion of bone healing (Roberts et al. 2023), and facilitation of wound healing (Panagiotou et al. 2023).

The cultivation of *Bifidobacterium* requires a variety of nutrients, including carbon and nitrogen sources, essential growth factors, and mineral elements, as well as an anaerobic environment (Dang et al. 2021). Although several studies have explored the high‐density cultivation of *Bifidobacterium*, the methods developed are often unsuitable for large‐scale production due to low cell yields (D. Wang, Wang, et al. 2018), complex procedures (Lu 2005), and high costs (Lv and Liang 2016). Additionally, significant strain‐specific differences exist among different strains (X. Gao et al. 2021). Therefore, it is necessary to optimize the culture medium and fermentation conditions for newly identified strains with probiotic potential. The response surface methodology, which integrates factorial design and regression analysis, is a collection of statistical techniques used to design experiments, build predictive models, evaluate the effects of multiple factors, and identify optimal conditions for achieving target outcomes (Dong et al. 2014). This method is particularly more suitable for multifactorial experimental designs. This method has been widely employed in the optimization of medium formulations and fermentation processes, yielding promising results across various applications (Guan et al. 2023; Zalila‐Kolsi et al. 2022; Falah et al. 2021; Ras El Gherab et al. 2019).

In this study, *B. longum* HSBL001 was selected as the research subject, and a modified de Mann–Rogosa–Sharpe (MRS) medium was used as the basal culture medium. The composition of the growth medium for *B. longum* HSBL001 was optimized using a series of experimental approaches, including single‐factor analysis, Plackett–Burman design (PBD), the method of steepest ascent, and central composite design (CCD) response surface methodology. Additionally, the fermentation process was optimized and validated in a 3 L fermentor to provide a reference framework for the industrial application and large‐scale production of *B. longum* HSBL001.

## 2 Results and Discussion

### 2.1 Optimization of Carbon and Nitrogen Sources

**Figure 1:**
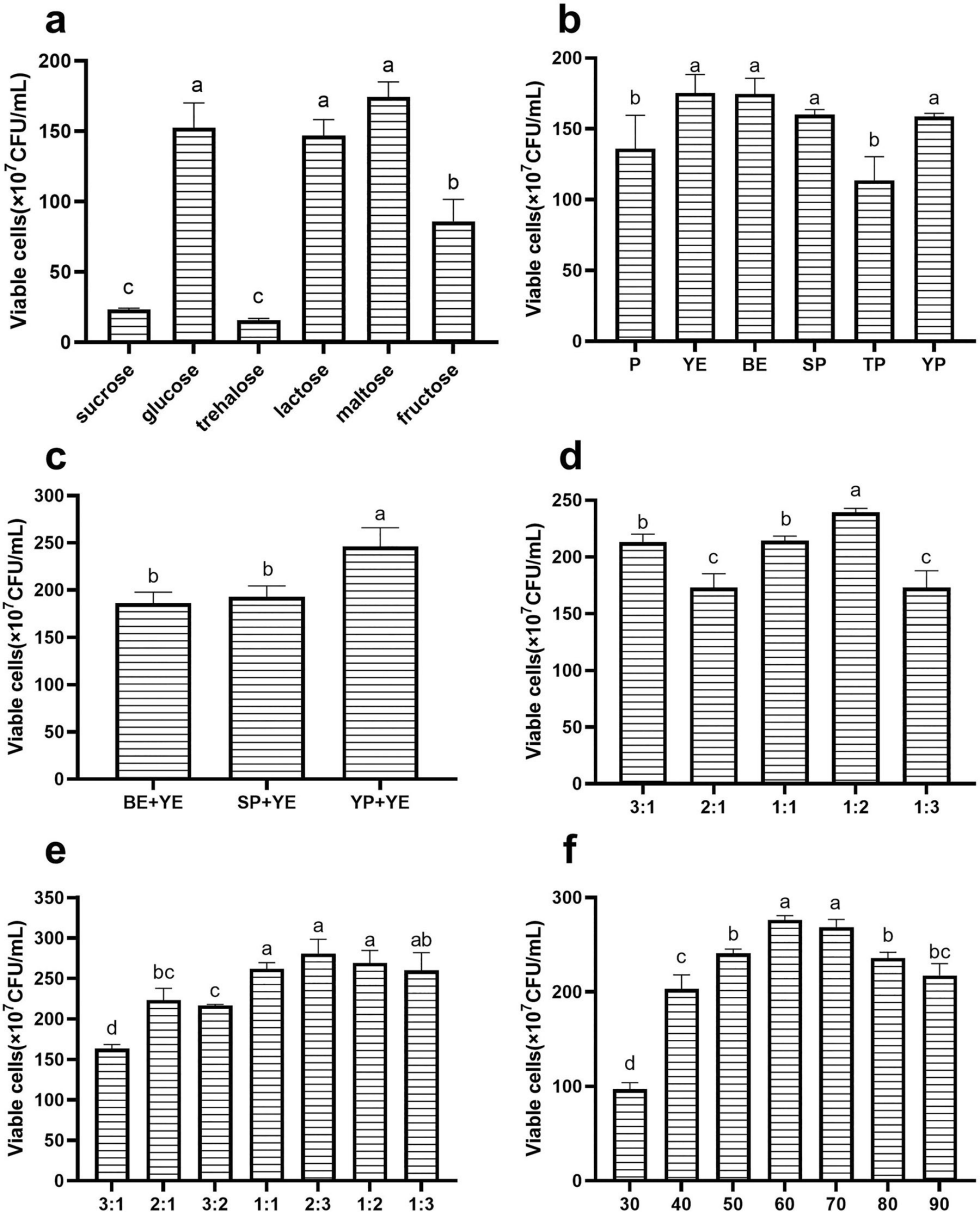
Effect of carbon source and nitrogen sources on the growth of *Bifidobacterium longum* HSBL001. (a) Type of carbon source, (b) type of nitrogen source, (c) compound nitrogen sources, (d) the proportion of YE/YP, (e) the proportion of carbon and nitrogen sources, and (f) the total amount of carbon and nitrogen sources. BE, beef extract; P, peptone; SP, soy peptone; TP, tryptone; YE, yeast extract; YP, yeast peptone.

The results of carbon source optimization are shown in Figure 1a. Among the tested carbon sources, maltose, glucose, and lactose showed a more favorable effect on the growth of *B. longum* HSBL001. The viable counts reached (1.745 ± 0.1061) × 10^9^ colony‐forming units (CFU/mL) for maltose, (1.525 ± 0.1768) × 10^9^ CFU/mL for glucose, and (1.47 ± 0.1131) × 10^9^ CFU/mL for lactose. As an essential nutrient for microbial growth, the carbon source plays a critical role in both biomass accumulation (Kundu et al. 2013) and the activity of microbial metabolites (Zhang et al. 2021). When maltose was used at a concentration of 20 g/L, the viable cell count was the highest. However, since no statistically significant difference was observed among the three carbon sources (*p* > 0.05), glucose was selected for subsequent medium optimization due to its lower cost.

The screening and combination of nitrogen sources are shown in Figure 1b–d. The viable cell counts, ranked from highest to lowest, were observed in media containing yeast extract, beef extract, yeast peptone, and soybean peptone. The combination of yeast extract and yeast peptone yielded significantly higher viable counts compared to other groups, reaching (2.46 ± 0.2007) × 10^9^ CFU/mL. When the ratio of yeast extract and yeast peptone was 1:2, the viable counts reached (2.3933 ± 0.0351) × 10^9^ CFU/mL. As a key nutritional substrate, the nitrogen source plays a vital role in the growth of lactic acid bacteria. Due to the presence of proteolytic enzymes, different lactic acid bacteria exhibit varying preferences for nitrogen sources (Sun et al. 2021). The ability of *B. longum* to utilize a nitrogen source depends on its richness in growth‐promoting factors, with a preference for yeast‐based nitrogen sources (X. Gao et al. 2021). Compared with single nitrogen sources, compound nitrogen sources are generally more favorable for microbial growth. However, the effect of combining yeast‐derived nitrogen with other types of nitrogen sources can vary depending on the specific strain.

The optimization results for the carbon‐to‐nitrogen ratio are shown in Figure 1e,f. The best growth performance was achieved at a carbon‐to‐nitrogen ratio of 2:3. When the total concentration of carbon and nitrogen reached 60 g/L, the highest viable count was observed, reaching (2.7633 ± 0.0451) × 10^9^ CFU/mL. The optimal carbon‐to‐nitrogen ratio varies among different *Bifidobacterium* strains (Yun et al. 2020). Maintaining a stable carbon source concentration, such as through carbon feeding based on alkali consumption, can significantly increase cell density (Cui et al. 2019). In addition, the optimal carbon‐to‐nitrogen ratio is equivalent to the consumption ratio at the point where the microbial growth rate begins to decline. At this stage, osmotic pressure can significantly affect cell growth and viability (Cui et al. 2021). Therefore, considering both cost efficiency and the influence of osmotic pressure, a total carbon and nitrogen concentration of 60 g/L was selected for subsequent optimization.

### 2.2 Optimization of Trace Elements and Growth Factors

**Figure 2:**
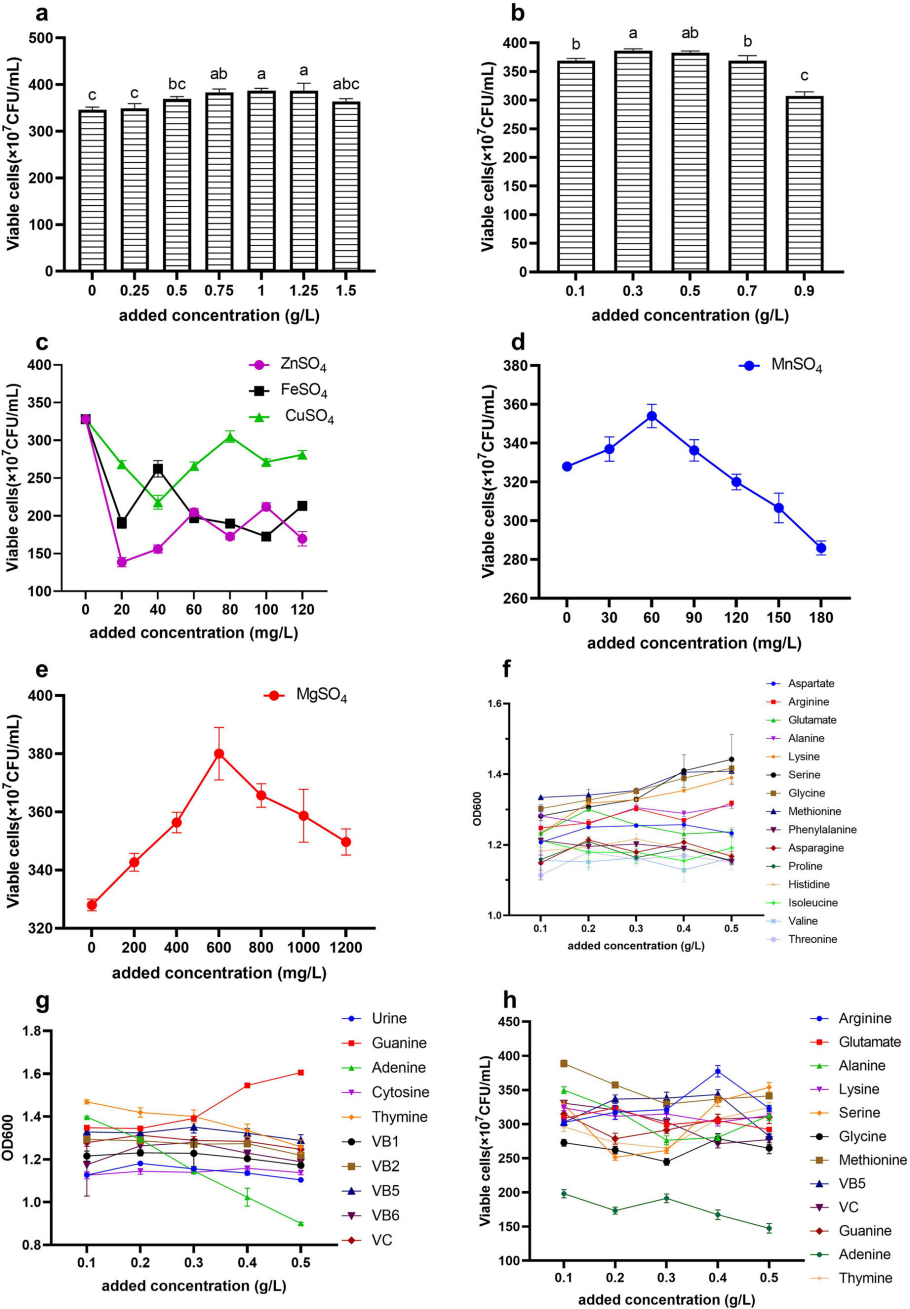
Effect of the trace elements and growth factors on the growth of *Bifidobacterium longum* HSBL001. (a) Tween‐80, (b) l‐cysteine hydrochloride, (c) ZnSO_4_, FeSO_4_, and CuSO_4_, (d) MnSO_4_, (e) MgSO_4_, (f) amino acid, (g) nucleotide and vitamin, and (h) selected growth factors.

The growth of *B. longum* HSBL001 initially increased and then decreased with increasing concentrations of Tween‐80 and l‐cysteine hydrochloride. As shown in Figure 2a,b, the optimal concentrations were 1 g/L for Tween‐80 and 0.3 g/L for l‐cysteine hydrochloride, resulting in viable counts of (3.27 ± 0.05) × 10^9^ CFU/mL and (3.26 ± 0.0346) × 10^9^ CFU/mL, respectively. The effects of metal ions on the growth of *B. longum* HSBL001 are shown in Figure 2c–e. Zinc sulfate, ferrous sulfate, and copper sulfate exhibited varying degrees of inhibitory effects on cell growth. In contrast, magnesium sulfate at 600 mg/L and manganese sulfate at 60 mg/L significantly promoted *B. longum* HSBL001 growth.

As a surfactant, Tween‐80 reduces cell agglutination and affects cell membrane permeability, thereby enhancing cell growth and promoting the extracellular release of metabolites (Y. Wang, Li, et al. 2018). l‐cysteine hydrochloride, a known reducing agent, lowers the redox potential of the fermentation broth and is thus considered a suitable additive for the cultivation of *Bifidobacterium*, which is highly sensitive to oxygen and hydrogen peroxide (Shu et al. 2013). Metal ions, serving as enzyme cofactors, can promote enzymatic reactions (X. Gao et al. 2021). Appropriate supplementation of trace elements can promote bacterial growth, whereas excessive amounts may exert toxic effects on bacterial cells (Boontun et al. 2021). Trace elements typically function as components of biologically active substances or serve as activators of enzymes.

Preliminary screening of growth factors based on cell density is shown in Figure 2f,g. The cell densities observed with the addition of arginine, glutamic acid, alanine, lysine, serine, glycine, methionine, guanine, adenine, thymine, vitamin B5, and vitamin C were higher than those of the control group. Therefore, viable cell counts for these selected growth factors were further evaluated, as shown in Figure 2h. The results indicated that specific concentrations of these compounds could enhance cell growth. Among them, 0.1 g/L methionine and 0.4 g/L arginine yielded significantly different viable counts, reaching (3.8867 ± 0.0814) × 10^9^ CFU/mL and (3.767 ± 0.0814) × 10^9^ CFU/mL, respectively.

*Bifidobacterium* is classified as a growth factor‐dependent heterotrophic microorganism. The addition of certain compounds, particularly those that are essential for regulating growth or metabolism but difficult for lactic acid bacteria to synthesize, can significantly enhance their growth (Yang et al. 2019). For example, tomato juice, which is rich in vitamins and minerals, has been shown to promote the growth of *Bifidobacterium*. The viable cell count of *Bifidobacterium* L‐DT reached 1.5 × 10^9^ CFU/mL with the addition of 3% tomato juice (Lu 2005). Similarly, hydrolysates of yak κ‐casein using different proteases served as nitrogen sources for *Bifidobacterium animalis* subsp. *lactis* BB12 and *B. longum* BBMN68, with amino acids such as alanine, methionine, tyrosine, phenylalanine, and arginine showing growth‐promoting effects (Tang et al. 2013), which aligns with our findings. Genome‐scale modeling has also revealed that methionine can serve as both the only amino acid and sulfur source for the growth of *B. longum* BB‐46 and *Bifidobacterium lactis* BB‐12 (Schöpping et al. 2021). Moreover, coenzyme A and pantothenic acid were found to promote growth in both strains, while vitamin B3 promoted only *Bifidobacterium lactis* BB‐12, and vitamin K was effective only for *B. longum* BB‐46. These findings underscore the strain‐specific requirements for growth factors. In this study, methionine and arginine were identified as the most effective compounds in promoting the growth of *B. longum* HSBL001.

### 2.3 PBD and Steepest Ascent Experiment

The Plackett–Burman experimental design and corresponding results are shown in Table 1. Nine factors were selected for evaluation: glucose (A), yeast extract (B), yeast peptone (C), magnesium sulfate heptahydrate (D), manganese sulfate monohydrate (E), Tween‐80 (F), l‐cysteine hydrochloride (G), methionine (H), and arginine (J). The results of the analysis of variance (ANOVA), shown in Table 2, indicate that the model is statistically significant, with a high correlation coefficient (*R* ^2^) of 0.9899, suggesting a strong correlation. Among the tested variables, yeast extract, yeast peptone, and arginine were identified as significant factors influencing cell growth. On the basis of predictions generated by Design‐Expert software, the optimized concentrations for the nonsignificant factors were as follows: glucose (27.36 g/L), manganese sulfate (0.09 g/L), magnesium sulfate (0.8 g/L), Tween‐80 (1 g/L), l‐cysteine hydrochloride (0.24 g/L), and methionine (0.15 g/L).

**Table 1:** Plackett–Burman experiment design and results.

Run order	A (g/L)	B (g/L)	C (g/L)	D (g/L)	E (g/L)	F (g/L)	G (g/L)	H (g/L)	J (g/L)	Actual viable cells (10^9^ CFU/mL)	Predicted viable cells (10^9^ CFU/mL)
1	1	1	−1	1	1	1	−1	−1	−1	3.52	3.4233
2	−1	1	1	−1	1	1	1	−1	−1	3.6133	3.71
3	1	−1	1	1	−1	1	1	1	−1	3.1667	3.1089
4	−1	1	−1	1	1	−1	1	1	1	3.7533	3.6956
5	−1	−1	1	−1	1	1	−1	1	1	3.94	3.8433
6	−1	−1	−1	1	−1	1	1	−1	1	2.48	2.5378
7	1	−1	−1	−1	1	−1	1	1	−1	1.7867	1.8444
8	1	1	−1	−1	−1	1	−1	1	1	3.1733	3.27
9	1	1	1	−1	−1	−1	1	−1	1	3.5333	3.4367
10	−1	1	1	1	−1	−1	−1	1	−1	3.6467	3.7044
11	1	−1	1	1	1	−1	−1	−1	1	3.6267	3.7233
12	−1	−1	−1	−1	−1	−1	−1	−1	−1	1.34	1.2822

*Note:* A, glucose; B, yeast extract; C, yeast peptone; D, manganese sulfate; E, magnesium sulfate; F, Tween‐80; G, l‐cysteine hydrochloride; H, methionine; J, arginine.

**Table 2:** Variance analysis of Plackett–Burman experiment design.

Source	Sum of squares	df	Mean square	*F* value	*p* value	Significance
Model	7.46	9	0.8294	21.8	0.0446	*
A	0.0001	1	0.0001	0.0024	0.9651	
B	2	1	2	52.59	0.0185	*
C	2.5	1	2.5	65.62	0.0149	*
D	0.6565	1	0.6565	17.26	0.0534	
E	0.7008	1	0.7008	18.42	0.0502	
F	0.4058	1	0.4058	10.67	0.0823	
G	0.0695	1	0.0695	1.83	0.309	
H	0.1526	1	0.1526	4.01	0.1831	
J	0.9822	1	0.9822	25.82	0.0366	*

*Note:* A, glucose; B, yeast extract; C, yeast peptone; D, manganese sulfate; E, magnesium sulfate; F, Tween‐80; G, l‐cysteine hydrochloride; H, methionine; J, arginine.

*R* ^2^ = 0.9899, *R* ^2^_Adj_ = 0.9445; CV = 6.23%, Adeq Precision = 14.3838.

* *p* < 0.05.

Table 2 shows that the sum of squares for yeast extract, yeast peptone, and arginine was all positive, indicating that their concentrations were positively correlated with cell growth in the PBD. Therefore, their levels were gradually increased in the steepest ascent experiment. As shown in Table 3, the fourth formulation yielded the highest viable cell count and was thus selected as the central point for the subsequent CCD experiment.

**Table 3:** Steepest ascent coordination path and result.

Step	B (g/L)	C (g/L)	J (g/L)	Viable cells (10^9^ CFU/mL)
Base	12	24	0.4	3.56
Base + *δ*	14	26	0.44	3.7467
Base + 2*δ*	16	28	0.48	3.88
Base + 3*δ*	18	30	0.52	4.1667
Base + 4*δ*	20	32	0.56	3.8267
Base + 5*δ*	22	34	0.6	3.58

*Note:* B, yeast extract; C, yeast peptone; J, arginine.

### 2.4 CCD Response Surface Experiment

On the basis of regression analysis of the experimental data, the quadratic polynomial regression equation describing the relationship between viable counts and the concentrations of yeast extract, yeast peptone, and arginine was derived as follows:

$$\begin{array}{ccl} Y & = & -5.4638+0.38508A+12.88438B+12.88438C\\ & & -0.00075\text{AB}-0.11083\text{AC}-0.12748\text{BC}\\ & & -0.00766A^{2}-0.00109B^{2}-6.19293C^{2}. \end{array}$$

As shown in Table 4, the model was highly significant (*p* < 0.01), and the lack‐of‐fit term was not significant (*p* > 0.05). On the basis of the *F* values of the model terms, the order of influence on cell proliferation was as follows: yeast extract > arginine > yeast peptone. The linear terms A, C, A^2^, and C^2^ had significant effects on cell proliferation. Interaction terms AC and BC also showed significant effects on cell proliferation, while the other terms were not statistically significant. The correlation coefficients *R* ^2^ and *R* ^2^_Adj_ were 0.9523 and 0.9094, respectively, indicating that the model reliably explains most of the variability in the response. The Adeq Precision of the model was 16.0420, which is well above the commonly accepted threshold of 4, indicating a strong signal‐to‐noise ratio and high model credibility. A coefficient of variation below 10% is generally considered acceptable, with lower values indicating higher test reliability. The coefficient of variation of this model was 1.81%, further confirming the reliability of the experimental results (QX Cheng and Xiong 2020).

**Table 4:** Variance analyses of the regression equation.

Source	Sum of squares	df	Mean square	*F* value	*p* value	Significance
Modol	0.9599	9	0.1067	22.1844	< 0.0001	**
A	0.2890	1	0.2890	60.1146	< 0.0001	**
B	0.0017	1	0.0017	0.3601	0.5618	
C	0.0533	1	0.0533	11.0836	0.0076	**
AB	0.0028	1	0.0028	0.5842	0.4623	
AC	0.0246	1	0.0246	5.1095	0.0473	*
BC	0.0325	1	0.0325	6.7601	0.0265	*
A2	0.5289	1	0.5289	110.0181	< 0.0001	**
B2	0.0106	1	0.0106	2.2137	0.1676	
C2	0.0553	1	0.0553	11.4966	0.0069	**
Residual	0.0481	10	0.0048			
Lack of fit	0.0329	5	0.0066	2.1629	0.2086	
Pure error	0.0152	5	0.0030			
Cor total	1.0080	19				

*Note:* A, yeast extract; B, yeast peptone; C, arginine; *R* ^2^ = 0.9523, *R* ^2^_Adj_ = 0.9094; CV = 1.81%; Adeq Precision = 16.0426.

* *p* < 0.05

** *p* < 0.01.

**Figure 3:**
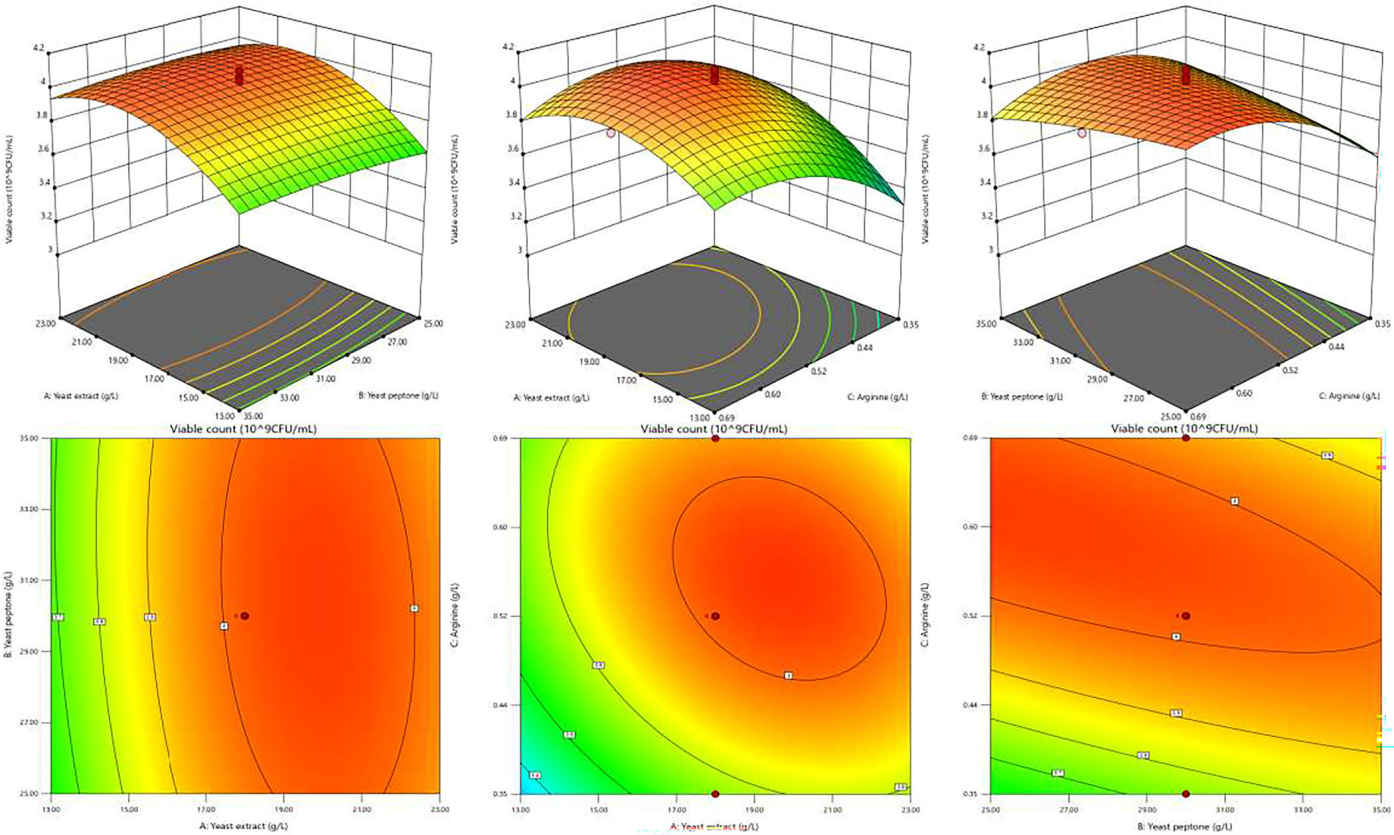
Effects of interaction among yeast extract, yeast peptone, and arginine on the cell density of *Bifidobacterium longum* HSBL001. a1, a2: Representing AB interaction when C is constant. b1, b2: Representing AC interaction when B is constant. c1, c2: Representing BC interaction when A is constant. Blue color indicates the lowest response viable cells count, while the red color shows the highest value of response viable cells count.

By maintaining one of the factors constant, the response surfaces illustrating the effects of the other two factors on viable cell counts were generated. The steeper the slope of the response surface, the more pronounced the influence of the factors on the response value. Moreover, an obliquely elliptical contour plot suggests significant interaction between the two factors (XY Cheng et al. 2023). The response surface plots and corresponding contour maps are shown in Figure 3. Viable cell counts initially increased and then decreased with increasing levels of each factor. Contour plots revealed a significant interaction between arginine (C) and both yeast extract (A) and yeast peptone (B) in influencing viable cell counts (*Y*) (*p* < 0.05).

On the basis of the regression model, the optimal concentrations for maximizing viable cell counts were determined as follows: yeast extract 19.524 g/L, yeast peptone 25.85 g/L, and arginine 0.599 g/L. The predicted viable count under these conditions was 4.06103 × 10^9^ CFU/mL. To validate the reliability of the model and optimization results, verification experiments were conducted, yielding an actual viable count of (3.93 ± 0.0794) × 10^9^ CFU/mL. This value represents a 2.58‐fold increase compared to the modified MRS medium. The close agreement between the predicted and experimental values confirms the reliability of the model, demonstrating that it can effectively predict the effect of medium composition on bacterial growth. Similar findings have been reported in recent studies. For example, the application of a Box–Behnken design for medium optimization increased the viable cell count of *Lactobacillus plantarum* Y44 by 6.11‐fold (Ding et al. 2023). Another study using a response surface methodology achieved a 107% increase in the cell dry weight of *L. plantarum* Pi06 (Hwang et al. 2012).

### 2.5 Optimization of Culture Condition

**Figure 4:**
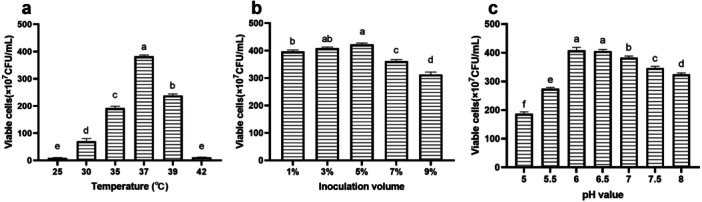
Effects of culture conditions on the growth of *Bifidobacterium longum* HSBL001. (a) Temperature, (b) inoculation volume, and (c) pH value.

The optimization results for fermentation parameters are shown in Figure 4. The viable cell count of *B. longum* HSBL001 initially increased and then decreased with increasing initial pH, inoculation size, and fermentation temperature. At an initial pH of 6, the highest viable cell count was observed, reaching (4.0833 ± 0.1041) × 10^9^ CFU/mL. An appropriate initial pH enhances cell membrane fluidity and maintains its integrity by increasing the degree of unsaturation of membrane fatty acids (Sun et al. 2022). The highest viable cell count of (4.2267 ± 0.0462) × 10^9^ CFU/mL was obtained at an inoculum size of 5% (v/v). Inoculum size significantly affects the entire fermentation process. A low inoculation level may extend the fermentation time, while excessive inoculation can result in rapid nutrient depletion, thereby shortening the logarithmic and stationary phases and affecting bacterial viability (YW Lin et al. 2017). The viable cell count reached (4.1867 ± 0.0808) × 10^9^ CFU/mL at 37°C, which was significantly higher than the counts observed at other temperatures. A recent study reported that the optimal cultivation temperature for *B. longum* ranges from 37°C to 42°C (D. Wang, Wang, et al. 2018). Temperature plays a critical role in regulating intracellular enzyme activity in cells. Deviations from the optimal range, either too high or too low, can impair metabolic functions and inhibit bacterial growth (Choi et al. 2021).

### 2.6 Comparison of Optimized and Modified MRS Medium in a Bioreactor

**Figure 5:**
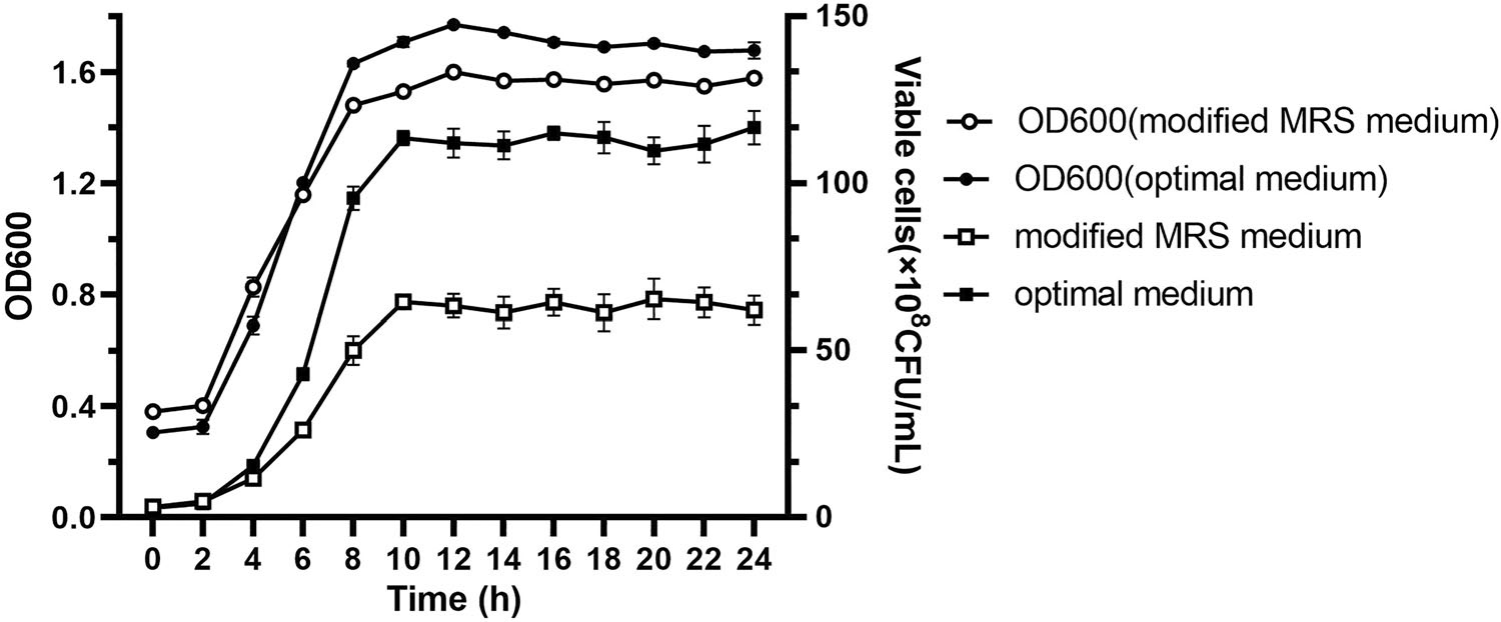
The growth curve of *Bifidobacterium longum* HSBL001 in modified MRS medium and optimized medium. MRS, de Mann–Rogosa–Sharpe.

The growth curve of *B. longum* HSBL001 cultured in modified MRS medium and then optimized medium under identical conditions (37°C, pH 6.0, 150 rpm, 5% inoculum) is shown in Figure 5. In both the modified MRS medium and the optimized formulation, the cell density of *Bifidobacterium* HSBL001 remained relatively constant from 0 to 2 h. A rapid increase in cell density was observed starting at 4 h, reaching maximum values of 1.6 and 1.77 at 12 h in the modified and optimized media, respectively, after which growth stabilized. These results indicate that the specific growth rate of *Bifidobacterium* HSBL001 was higher in the optimized medium compared to the modified MRS medium. As shown in Figure 5, both media exhibited a lag phase from 0 to 4 h, an exponential growth phase from 4 to 10 h, and a stationary phase after 10 h. The viable cell count in the modified MRS medium reached (6.53 ± 0.61) × 10^9^ CFU/mL at 20 h, whereas the optimized formulation achieved (1.17 ± 0.05) × 10^10^ CFU/mL at 24 h, representing a 1.786‐fold increase over the modified MRS medium.

Three strains of *B. longum* have been reported to achieve high cell densities using different concentrations of glucose, yeast extract, and MgSO_4_ as medium components. Under fermentation conditions of 5% inoculum and a constant pH of 5.0, the final viable cell counts in the fermentation broth reached 0.79 × 10^10^, 0.95 × 10^10^, and 1.1 × 10^10^ CFU/mL, respectively (X. Gao et al. 2021). Similarly, *L. plantarum* 200655 achieved a viable count of 9.56 log CFU/mL in MRS medium, while the optimized medium formula increased this to 10.03 log CFU/mL (YW Lin et al. 2017). Currently, industrial‐scale production primarily relies on pH‐controlled batch fermentation. Higher fermentation efficiency can be achieved by maintaining an optimal carbon‐to‐nitrogen ratio and mitigating osmotic stress during the process (Zhang et al. 2022). Furthermore, continuous fermentation with an integrated membrane filtration system has been shown to enhance nutrient utilization, biomass, and metabolite production (Mende et al. 2012). However, this approach carries a higher risk of microbial contamination. Future advancements may focus on the application of targeted nutritional supplementation to further improve fermentation performance.

## 3 Conclusion

In this study, the culture medium composition and fermentation conditions for *B. longum* HSBL001 were systematically optimized. The resulting optimal medium formulation consisted of yeast extract (19.524 g/L), yeast peptone (25.85 g/L), arginine (0.599 g/L), glucose (27.36 g/L), manganese sulfate (0.09 g/L), magnesium sulfate (0.8 g/L), Tween‐80 (1 g/L), l‐cysteine (0.24 g/L), methionine (0.15 g/L), potassium dihydrogen phosphate (2 g/L), sodium acetate (4 g/L), and ammonium citrate (2 g/L). The optimal culture conditions included a 5% inoculum size, fermentation temperature of 37°C, and an initial pH of 6.0, under which a viable cell count of 4.20 × 10^9^ CFU/mL was achieved in shaking flask fermentation. When scaled up to a 3 L bioreactor, the viable cell count reached 1.17 × 10^10^ CFU/mL, representing a 1.786‐fold increase compared to the modified MRS medium. These results showed that the optimized medium and fermentation strategy significantly enhance the growth of *B. longum* HSBL001, providing a valuable reference for its future industrial‐scale production.

## 4 Materials and Methods

### 4.1 Microbial Strain and Medium

*B. longum* HSBL001, originally isolated from the feces of healthy infants, has been used commercially in the probiotic formulation BIFIDO (Jincheng Health Pharmaceutical Co. Ltd., Jincheng, China) for over 20 years (Fu Huiling and Wei 2002; Bai Xiaoru et al. 2003). The strain was preserved in glycerol cryovials at −80°C and revived on MRS agar supplemented with 0.05% l‐cysteine hydrochloride. Cultures were incubated anaerobically at 37°C for 48 −72 h. A single colony was then selected and transferred into modified MRS broth for 24 h. This activation procedure was repeated twice to obtain a viable working culture for subsequent experiments (Averina et al. 2023). Fermentation conditions for the preculture were as follows: initial pH of 7.0, inoculum size of 2%, and anaerobic incubation at 37°C for 18 h. All culture operations were conducted in an anaerobic workstation (A35 Anaero Workstation, Don Whitley Scientific Ltd., UK) to ensure strict anaerobic conditions.

### 4.2 Single‐Factor Optimization of Medium Composition

Single‐factor experiments were conducted to optimize the composition of the growth medium, including carbon and nitrogen sources, their ratio and total concentration, Tween‐80, l‐cysteine hydrochloride, trace elements, and growth factors. All experiments were carried out in a 50 mL fermentation system. To determine the optimal carbon source, six carbohydrates, sucrose, glucose, lactose, fructose, maltose, and trehalose, were each added at a concentration of 20 g/L. For nitrogen source optimization, 25 g/L of each of the following was tested: peptone, soybean peptone, yeast extract, beef extract powder, casein peptone, and tryptone. The best‐performing nitrogen source was then combined at a 1:1 ratio with the others to identify the most effective nitrogen source combination. Next, the optimal mixing ratio of compound nitrogen sources was evaluated using seven proportions: 1:3, 1:2, 2:3, 1:1, 3:2, 2:1, and 3:1. Keeping the total carbon and nitrogen concentration constant at 60 g/L, single‐factor experiments were conducted to determine the best carbon‐to‐nitrogen ratio using the same set of proportions. Finally, the total concentrations of carbon and nitrogen were tested at 40, 50, 60, 70, 80, and 90 g/L to identify the ideal overall nutrient composition. On the basis of these results, Tween‐80 was tested at concentrations of 0, 0.25, 0.5, 0.75, 1, 1.25, and 1.5 g/L, while l‐cysteine hydrochloride was tested at 0.1, 0.3, 0.5, 0.7, and 0.9 g/L to determine the optimal levels of these additives.

Trace element optimization was performed by evaluating the viable cell counts in a culture medium supplemented with different concentrations of zinc sulfate heptahydrate, ferric sulfate heptahydrate, copper sulfate pentahydrate, manganese sulfate monohydrate, and magnesium sulfate heptahydrate. To identify suitable factors, the OD600 was measured in media containing 15 amino acids (aspartic acid, arginine, glutamic acid, alanine, lysine, serine, glycine, methionine, phenylalanine, asparagine, proline, histidine, isoleucine, valine, and threonine), five nucleotides (uracil, guanine, adenine, cytosine, and thymine), and five vitamins (VB1, VB2, VB5, VB6, and VC). Final selections and concentrations of optimal growth factors were then determined based on viable cell counts.

### 4.3 PBD and Steepest Ascent Method

On the basis of the results of the preliminary single‐factor optimization experiments, a PBD was employed to identify the most significant factors influencing the growth of *B. longum* HSBL001. The variables selected for evaluation included glucose (A), yeast extract (B), yeast peptone (C), magnesium sulfate heptahydrate (D), manganese sulfate monohydrate (E), Tween‐80 (F), l‐cysteine hydrochloride (G), methionine (H), and arginine (J). Each factor was tested at two levels, coded as −1 (low) and +1 (high). The left side of the design matrix represents the low level (−1), while the right side represents the high level (+1). The specific values and coding of each variable are presented in Table 5. Following the PB screening, the factors with statistically significant effects were identified based on their regression coefficients. These selected variables were then subjected to the steepest ascent method to determine the optimal direction and magnitude for increasing the response value. This process facilitated the identification of the region near the optimal response and provided the central point for the subsequent CCD experiments. The design and results of the steepest ascent experiment are shown in Table 6.

**Table 5:** Factors and levels of Plackett–Burman design.

Factors	Coding	Low level (−1) (g/L)	High level (+1) (g/L)
Glucose	A	19	29
Yeast extract	B	7	17
Yeast peptone	C	19	29
Magnesium sulfate heptahydrate	D	0.03	0.09
Manganese sulfate monohydrate	E	0.4	0.8
Tween‐80	F	0.5	1
l‐cysteine hydrochloride	G	0.2	0.4
Methionine	H	0.05	0.15
Arginine	J	0.3	0.5

*Note:* A, glucose; B, yeast extract; C, yeast peptone; D, manganese sulfate; E, magnesium sulfate; F, Tween‐80; G, l‐cysteine hydrochloride; H, methionine; J, arginine.

**Table 6:** Steepest ascent method design.

Step	B	C	J
Base	12	24	0.4
*δ*	2	2	0.04
Base + *δ*	14	26	0.44
Base + 2*δ*	16	28	0.48
Base + 3*δ*	18	30	0.52
Base + 4*δ*	20	32	0.56
Base + 5*δ*	22	34	0.6

*Note:* B, yeast extract; C, yeast peptone; J, arginine.

### 4.4 Response Surface Methodology

On the basis of the previous PBD and the steepest ascent method, the central point for a CCD was determined. CCD was employed to further optimize the medium composition by exploring the interactions and quadratic effects of the key variables. Using Design‐Expert software, a CCD matrix was constructed, incorporating three significant factors at five coded levels each, as shown in Table 7. The experimental design consisted of 14 factorial and axial points, along with six replicates at the center point, to estimate experimental error, yielding a total of 20 experimental runs (Table 8). Regression analysis of the test results obtained from the CCD was performed and fitted to a quadratic polynomial model to predict the optimal medium formulation.

**Table 7:** Factors range of central composite design experiments.

Factors	Unit	Level				
		−1.68179	−1	0	1	1.68179
Yeast extract	g/L	9.59	13	18	23	26.41
Yeast peptone	g/L	21.59	25	30	35	38.41
Arginine	g/L	0.35	0.42	0.52	0.62	0.69

**Table 8:** Experimental design and results for response surface analysis.

Run	A (g/L)	B (g/L)	C (g/L)	Actual viable cells (10^9^ CFU/mL)	Predicted viable cells (10^9^ CFU/mL)
1	−1	−1	−1	3.4467	3.145
2	1	−1	−1	3.78	3.6598
3	−1	1	−1	3.6033	3.4194
4	1	1	−1	3.93	3.8594
5	−1	−1	1	3.78	3.756
6	1	−1	1	3.96	3.898
7	−1	1	1	3.75	3.6016
8	1	1	1	3.7867	3.6688
9	−1.68179	0	0	3.1233	3.233374742
10	1.68179	0	0	3.7833	3.722775632
11	0	−1.68179	0	3.9033	3.924062814
12	0	1.68179	0	3.9333	3.962071268
13	0	0	−1.68179	3.72	3.347705107
14	0	0	1.68179	3.92	3.701217365
15	0	0	0	4.06	4.02
16	0	0	0	4.10	4.02
17	0	0	0	4.04	4.02
18	0	0	0	3.98	4.02
19	0	0	0	3.96	4.02
20	0	0	0	3.98	4.02

*Note:* A, yeast extract; B, yeast peptone; C, arginine.

### 4.5 Effects of Fermentation Conditions

To determine the optimal culture conditions for *B. longum* HSBL001, the effects of initial pH, fermentation temperature, and inoculation amount on viable cell counts were systematically evaluated. The initial pH of the culture medium was adjusted to values ranging from 5.0 to 8.0 using 1 M HCl or 1 M NaOH. Fermentation temperatures were set between 27°C and 42°C. Inoculation levels were set between 1% and 9% (v/v). Each fermentation trial was conducted under anaerobic conditions for 18 h, and the viable cell counts were measured to identify the optimal combination of fermentation parameters.

### 4.6 Scale‐Up Fermentation of Optimized Medium

Scale‐up fermentation of *B. longum* HSBL001 using the optimized medium and conditions was performed in a 3 L bioreactor with a working volume of 2 L. The fermentation medium was inoculated with 5% (v/v) of seed culture, previously grown in the optimized medium for 18 h. The temperature was controlled at 37°C, and the pH was maintained at 6.0 by automated addition of 1 M NaOH. The agitation speed was set to 150 rpm. To ensure strict anaerobic conditions, nitrogen gas was continuously sparged to remove residual oxygen and maintain a vessel pressure of 0.05 MPa. Fermentation using the modified MRS medium was conducted in parallel under identical conditions as a control. OD600 and viable cell counts were measured every 2 h over 24 h.

### 4.7 Assay of Cell Density (OD600) and Viable Cell Counts

The optical density of *B. longum* HSBL001 fermentation broth was measured using a multimode microplate reader (EnSight, PerkinElmer Ltd., USA) at a wavelength of 600 nm. The uninoculated medium served as the blank control. For viable cell enumeration, fermentation broth samples were serially diluted in sterile phosphate‐buffered saline, and appropriate dilutions were plated using the pour plate method on modified MRS agar. The plates were incubated anaerobically at 37°C for 48 h, after which CFU/mL were calculated.

### 4.8 Statistical Analysis

All experiments were performed independently in triplicate, and the results are presented as mean ± standard error (SE). PBD and CCD were implemented using Design‐Expert software (version 13) for experimental design and regression analysis. ANOVA was performed using Jamovi software (version 2.4.8). A *p* value of less than 0.05 was considered statistically significant.

## Author Contributions

**Hao Cheng:** conceptualization (lead), data curation (equal), formal analysis (lead), investigation (equal), methodology (lead), writing – original draft (lead), writing – review and editing (equal). **Jiangbin Liu:** formal analysis (equal), investigation (equal), visualization (equal), writing – original draft (supporting). **Liya Mei:** investigation (equal), writing – original draft (equal). **Wei Liu:** investigation (equal), validation (equal). **Fengxi Yang:** investigation (equal), validation (equal). **Xiaojuan Ma:** investigation (equal). **Yan Zhang:** investigation (equal). **Youfa Xie:** supervision (equal), writing – review and editing (equal). **Yang Zhang:** supervision (equal), writing – review and editing (equal). **Yanxia Xiong:** supervision (equal), writing – review and editing (equal).

## Conflicts of Interest

None declared.

## Data Availability

All data generated or analyzed during this study are included in this published article.
